# A bladder cancer microenvironment simulation system based on a microfluidic co-culture model

**DOI:** 10.18632/oncotarget.6070

**Published:** 2015-10-10

**Authors:** Peng-fei Liu, Yan-wei Cao, Shu-dong Zhang, Yang Zhao, Xiao-guang Liu, Hao-qing Shi, Ke-yao Hu, Guan-qun Zhu, Bo Ma, Hai-tao Niu

**Affiliations:** ^1^ Department of Urology, The Affiliated Hospital of Qingdao University, Qingdao, Shandong, China; ^2^ The Medical College of Qingdao University, Qingdao, Shandong, China; ^3^ Key Urology Laboratory of Qingdao City, Qingdao, Shandong, China; ^4^ Department of Urology, Peking University Third Hospital, Beijing, China; ^5^ Single Cell Center, CAS Key Laboratory of Biofuels and Shandong Key Laboratory of Energy Genetics, Qingdao Institute of Bioenergy and Bioprocess Technology, Chinese Academy of Sciences, Qingdao, Shandong, China

**Keywords:** microfluidic device, bladder cancer, microenvironment, three-dimensional culture, co-culture, drug sensitivity

## Abstract

A tumor microenvironment may promote tumor metastasis and progression through the dynamic interplay between neoplastic cells and stromal cells. In this work, the most representative and significant stromal cells, fibroblasts, endothelial cells, and macrophages were used as vital component elements and combined with bladder cancer cells to construct a bladder cancer microenvironment simulation system. This is the first report to explore bladder cancer microenvironments based on 4 types of cells co-cultured simultaneously. This simulation system comprises perfusion equipment, matrigel channel units, a medium channel and four indirect contact culture chambers, allowing four types of cells to simultaneously interact through soluble biological factors and metabolites. With this system, bladder cancer cells (T24) with a tendency to form a ‘reticular’ structure under 3 dimensional culture conditions were observed in real time. The microenvironment characteristics of paracrine interactions and cell motility were successfully simulated in this system. The phenotype change process in stromal cells was successfully reproduced in this system by testing the macrophage effector molecule Arg-1. Arg-1 was highly expressed in the simulated tumor microenvironment group. To develop “precision medicine” in bladder cancer therapy, bladder cancer cells were treated with different clinical ‘neo-adjuvant’ chemotherapy schemes in this system, and their sensitivity differences were fully reflected. This work provides a preliminary foundation for neo-adjuvant chemotherapy in bladder cancer, a theoretical foundation for tumor microenvironment simulation and promotes individual therapy in bladder cancer patients.

## INTRODUCTION

Bladder cancer is one of the most common malignant urologic diseases with high mortality and ranks eleventh in morbidity among human tumors worldwide.[1] Currently, many cell-based assays for tumor cells have been performed in monoculture or a two-dimensional cell culture mode.[2] Neglecting a tumor cell's microenvironment is a limitation of such research.

A tumor microenvironment is pivotal for mutationally corrupted cell proliferation, progression, and metastasis and can determine the malignant phenotype of bladder cancer.[3] A tumor microenvironment consists of tumor cells, non-neoplastic cells and an extracellular matrix. Resident and recruited cells are crucial non-neoplastic cells for the tumor microenvironment and are mainly composed of endothelial cells, fibroblasts, macrophages and other inflammatory cells. The interplay between tumor cells and the surrounding non-neoplastic cells stimulates the vital processes angiogenesis, invasion and immunosurveillance in solid tumor cells. [4, 5] Endothelial cells are a major cellular component of tumor vessels. The interaction between cancer cells and endothelial cells is an essential part of cancer cell intravasation migration through crossing endothelial barriers.[6] Fibroblasts and macrophages are the largest groups of non-neoplastic cells and inflammatory cells, respectively. Paracrine interactions between tumor cells and non-neoplastic cells are one of the driving forces of fibroblast and macrophage phenotypic alterations, which are referred to as cancer-associated-fibroblasts (CAFs) and tumor-associated-macrophages (TAM). Recent research shows that many factors secreted by tumor cells can facilitate the transformed phenotype of fibroblasts, such as PDGF-α/β and bFGF. Several factors released from CAFs, such as HGF and CXCL14, can promote tumor cell progression.[7] In addition, the microenvironment determines drug sensitivity for tumor cells. Macrophages, fibroblasts and endothelial cells are vital components of a tumor microenvironment and enhance tumor drug resistance in tumors.[8-12]

Microfluidic technology is characterized by the engineered manipulation of fluids at the submillimeter scale and provides a promising approach for studying tumor microenvironments. Several studies have used microfluidic technology to analyze the biological characteristics of tumor microenvironments. Hyunjae Lee et al. reported on a metastatic microfluidic device to quantitatively assay cancer angiogenesis and transendothelial migration.[13] Tsi-Hsuan Hsu et al. analyzed the paracrine loop between fibroblasts and lung cancer cells and verified that cancer cells can stimulate fibroblasts into myofibroblasts by releasing cytokines. [14] In those studies, the analysis of cancer cell biological characteristics focused on one type of stromal cell, which was a limitation of the research.

Based on the above considerations and to promote development of “precision medicine” in bladder cancer therapy, we constructed a bladder microenvironment simulation system based on microfluidic technology. This is the first report to explore bladder cancer microenvironments based on 4 types of cells co-cultured simultaneously, which introduces more biological elements into the microenvironment compared with a monoculture. In this research, the most representative and significant stromal cells, fibroblasts, endothelial cells, and macrophages were selected as component elements and combined with bladder cancer cells to reconstruct the microenvironment. Using this simulation system, the microenvironment characteristics of paracrine interactions and cell motility were successfully simulated. In addition, by testing the sensitivity of neo-adjuvant chemotherapy schemes for bladder cancer cells in this system, a preliminary theoretical foundation was generated for the individual therapy of bladder cancer.

## RESULTS

### Microfluidic device fabrication

**Figure 1:**
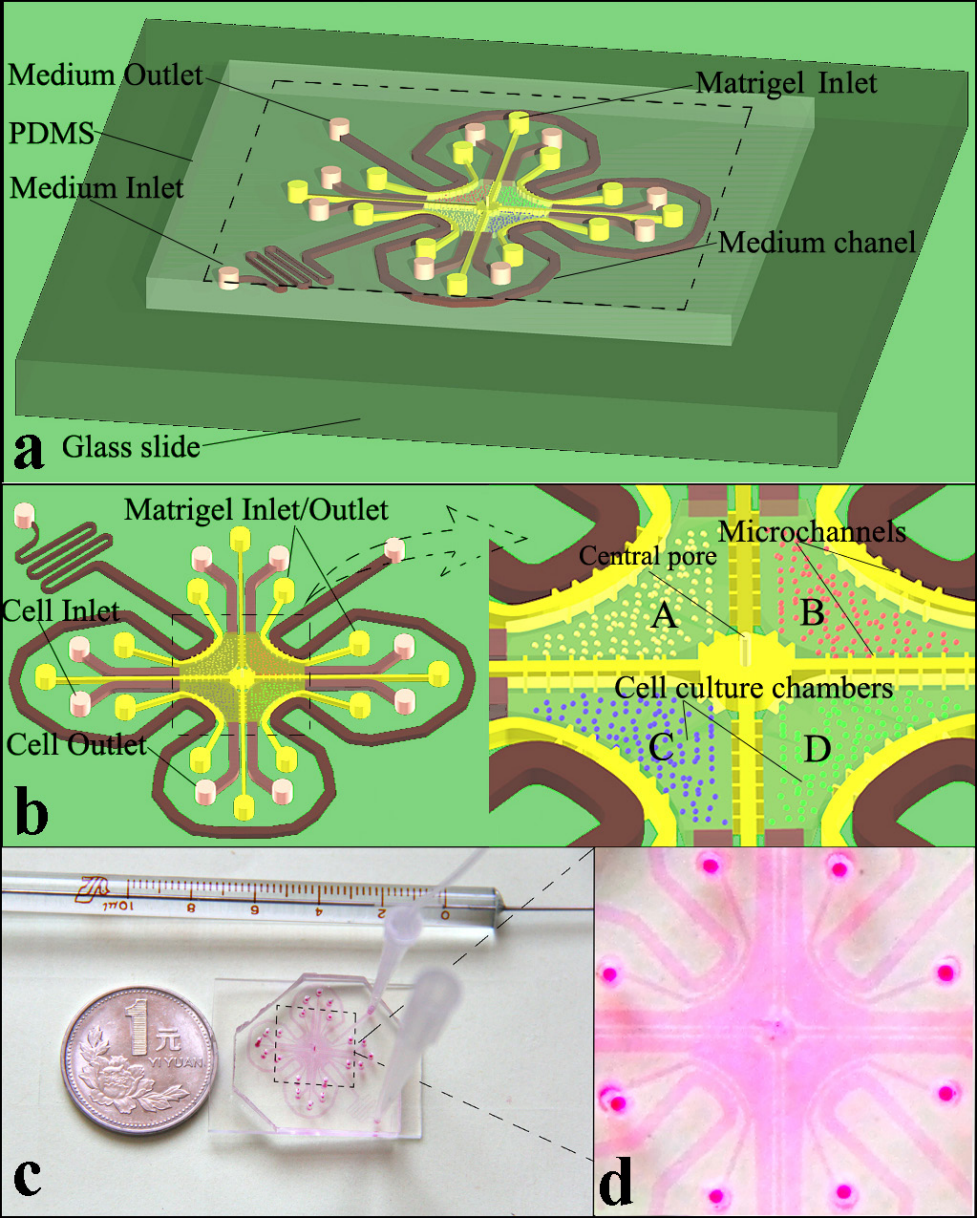
Microfluidic device blueprint and prototype **a.** Whole structure schematic illustration of the device. **b.** Magnified illustrations of the device structure. **c.** The microfluidic device connected to the perfusion equipment. **d.** Magnified section of the prototype.

The microfluidic device consists of four indirectly connected cell culture chambers (A, B, C, and D), matrigel channel units, and medium channels (Figure 1a). Cell inlet and outlet channels (pink) were connected to chambers that contain 7 shores (0.5-mm diameter) to avoid chamber collapse. The matrigel channel units (yellow) were composed of 4 uniform U-shaped matrigel channels (width = 200 μm, height = 50 μm) and a cross-shaped channel (width = 200 μm, height = 50 μm), which were located outside and inside of the co-culture chambers, respectively (Figure 1b). The medium channel and cell culture chamber were connected by the U-shaped matrigel channel, and the U-shaped matrigel channel was connected to 6 pairs of lateral microchannels (width = 50 μm, length = 100 μm, height = 50 μm) for medium spreading. For the cross-shaped matrigel channel units, 4 straight channels were connected to a central pool, which was located in the center of the device (Figure 1b). Each straight matrigel channel connected with 7 pairs of lateral microchannels (width = 50 μm, length = 100 μm, height = 50 μm) was connected to the adjacent cell co-culture chambers to maintain cytokine spreading from one cell culture chamber to others. The central pool (1.2 mm diameter) with a pore (0.5 mm diameter) was connected to 4 cell culture chambers by 4 pairs of microchannels (width = 50 μm, length = 150 μm, height = 50 μm) and performed a pressure balance function in the matrigel perfusion process (Figure 1b). The medium channel was composed of a medium inlet/outlet, U-shaped channels and a winding channel. As shown in Figure 1(c), the liquid column and microscale vacuum suction apparatus were connected to the inlet and outlet of the medium channel and formed a perfusion system. During the culture medium perfusion process, the microscale vacuum suction apparatus acted as a “trigger” to introduce the cell culture medium that filled the medium channel from the inlet to outlet. Next, removing the microscale vacuum suction apparatus from the outlet of this microfluidic device, the culture medium flowed slowly, relying on the liquid pressure of the liquid column.

### Validation of the microfluidic device

**Figure 2:**
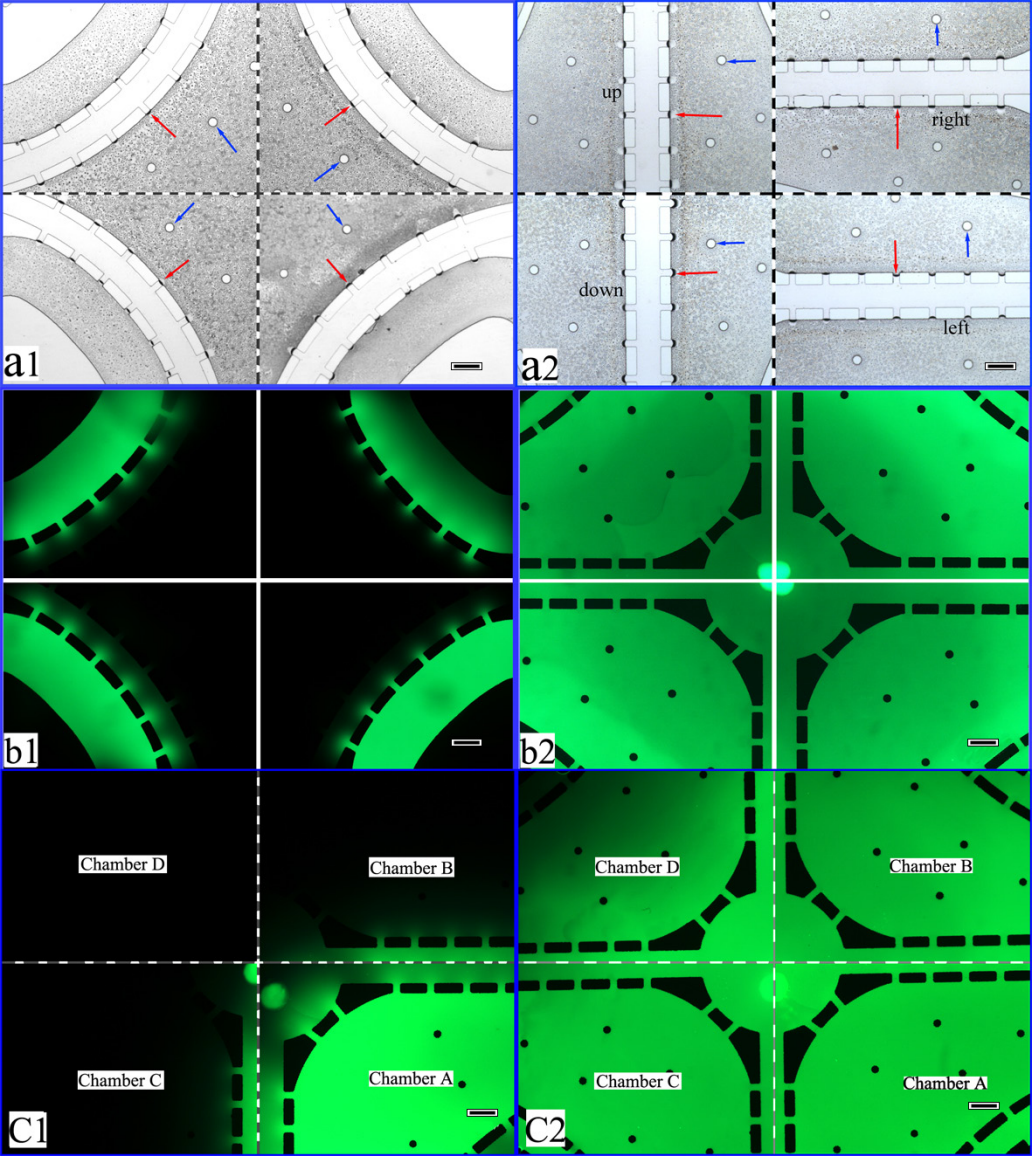
Performance validation of the co-cultural microfluidic device **a.** The matrigel perfusing process. The red arrow shows the interface of the matrigel. The blue arrow indicates the shores located in the cell culture chambers. (a1. The U-shaped matrigel channel filled with matrigel. a2. The straight matrigel channel filled with matrigel.) **b.** Diffusion of FITC-Dextran in the medium channel (b1. 10 min. b2. 180 min). **c.** Diffusion of FITC-Dextran in the chambers (c1. 10 min. c2. 200 min). For the microscopy images, 40×, scale bar 200 μm.

To test diffusion effectiveness for biological factors or soluble nutrients using this microfluidic device, FITC-Dextran was diffused on this microfluidic platform to simulate the diffusion process for biological factors and soluble nutrients. The matrigel perfusion performance of this system fully satisfied our design requirements. As shown in Figure 2a, the matrigel filled the matrigel channels and lateral channels simultaneously, but it did not spill into the medium channels and cell culture chambers. The U-shaped and cross-shaped channels were filled with matrigel as shown in Figure 2(a1) and Figure 2(a2), respectively. After the chambers were filled with the matrigel matrix (four-fold diluted with culture medium), FITC-Dextran was loaded into the medium channel and diffused from the medium channels to the cell culture chambers. The diffusion is shown in Figure 2. The FITC-Dextran diffusion microscopy images were collected at 10 min (Figure 2b1) and 180 min (Figure 2b2) after loading began. Diffusion in the chambers is shown in Figure 2C. After introducing the matrigel matrix (four-fold diluted with culture medium) into chambers B, C and D (Figure 2 c1), FITC-Dextran was loaded into chamber A. By contrasting the microscopy images captured at 10 min (Figure 2c1) and 200 min (Figure 2c2), diffusion in the chambers fully met the diffusion requirements. The diffusion process demonstrated that the system parameters fully satisfy the biological factor and soluble nutrient diffusion requirements.

### Cell morphological observations

**Figure 3:**
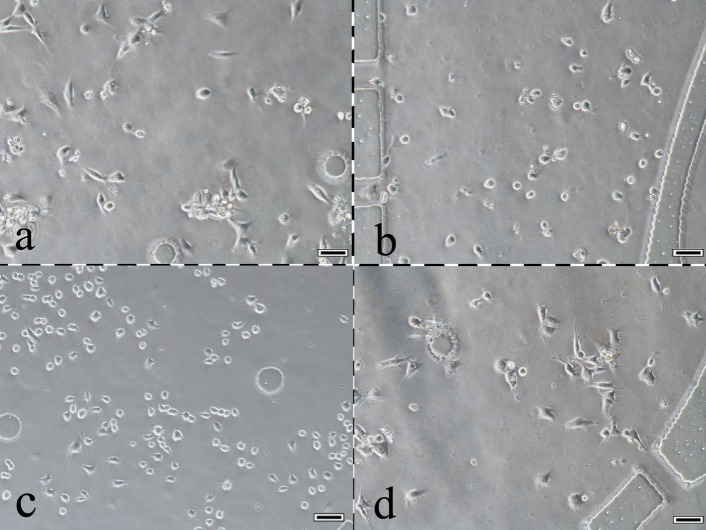
A photograph of cells growing in the 4 chambers of this system **a.** Fibroblasts. **b.** Bladder cancer cells. **c.** Macrophages. **d.** Endothelial cells. 100× scale bar 50 μm.

**Figure 4:**
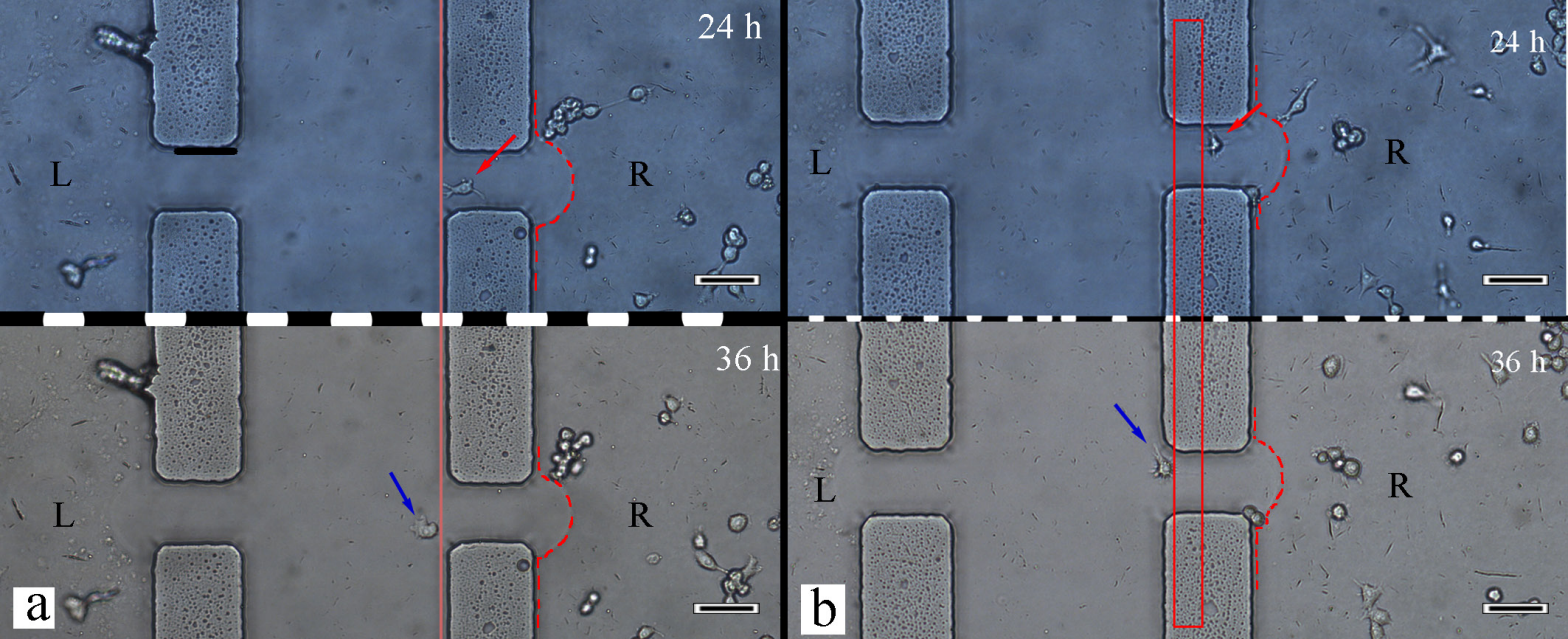
The macrophage cell migration in different lateral microchannels **a.** One lateral microchannel. **b.** Another lateral microchannel. The red dashed lines depict the matrigel interface. The red solid lines show the location changes of the same macrophage. The red/blue arrow indicates the location of the same macrophage after 24/36 hours of culture. L/R refers to the left/right chamber. The bladder cancer cells /macrophages are located in the L/R chamber, respectively. 100× scale bar 50 μm.

After culturing four types of cells for 12 h in this system, the cell morphous was captured by an inverted microscope. A portion of the cells exhibited a migration tendency through lateral microchannels in the matrigel matrix (Figure 3 and Figure 4). Four types of cells, fibroblasts, bladder cancer cells, macrophages, and endothelial cells, grew in four chambers and are shown in Figure 3(a-d), respectively. Observing the same cell (the arrow pointing), a macrophage migrated towards T24 cells in lateral microchannels through the matrigel matrix. The same macrophage at different locations was captured at 24/36 hours. The up/down image of the black dashed line in Figure 4 shows the micrographs captured at 24/36 hours, respectively.

**Figure 5:**
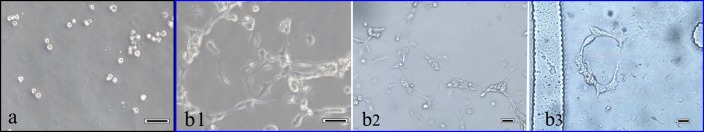
Photograph of T24 cells forming a ‘reticular’ structure **a.** After seeding for 5 min. b1, b2 and b3 after culturing for 18 hours. a, b1: 100×; b2, b3: 200× scale bar 50 μm.

In this research, the ‘reticular’ structure was formed during T24 cell proliferation under 3D culture conditions. The micrograph of the T24 cells after introduction into the culture chamber at 5 min is shown in Figure 5a. After culturing for 18 hours, the bladder cancer (T24) cells formed a remarkable ‘reticular’ structure in this system (Figure 5 b(1-3)).

### Arg-1 expression in macrophages in this system

**Figure 6:**
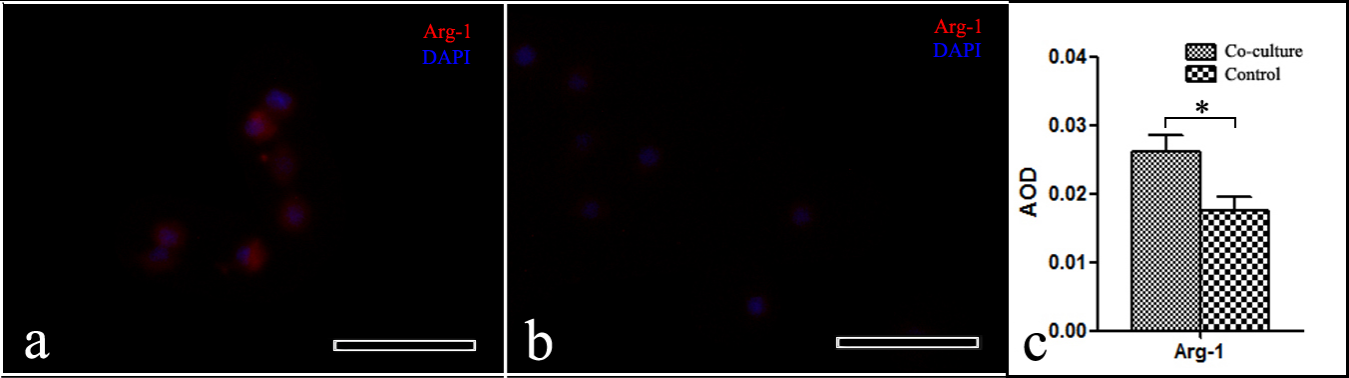
Photograph of Arg-1 expression in macrophages **a.** Microenvironment simulation group. **b.** Control group. 200×, scale bar: 50 μm. **c.** A pictograph of the average optical density of the macrophages, mean±SD, *p < 0.05.

An immunofluorescence staining assay was employed to detect the different levels of Arg-1 expression between the microenvironment simulation group and control group. The Arg-1 expression is shown in Figure 6. The red/blue immunofluorescence in the figure shows the Arg-1/nucleus in macrophages. Image J software was used to calculate the average optical density (AOD) of the micrographs. The red immunofluorescence (Arg-1) intensity in the macrophages from the microenvironment simulation group was stronger than in the control. The high Arg-1 macrophage expression in the tumor microenvironment was highly reproducible using this system.

### Bladder cancer cell death assessment

**Figure 7:**
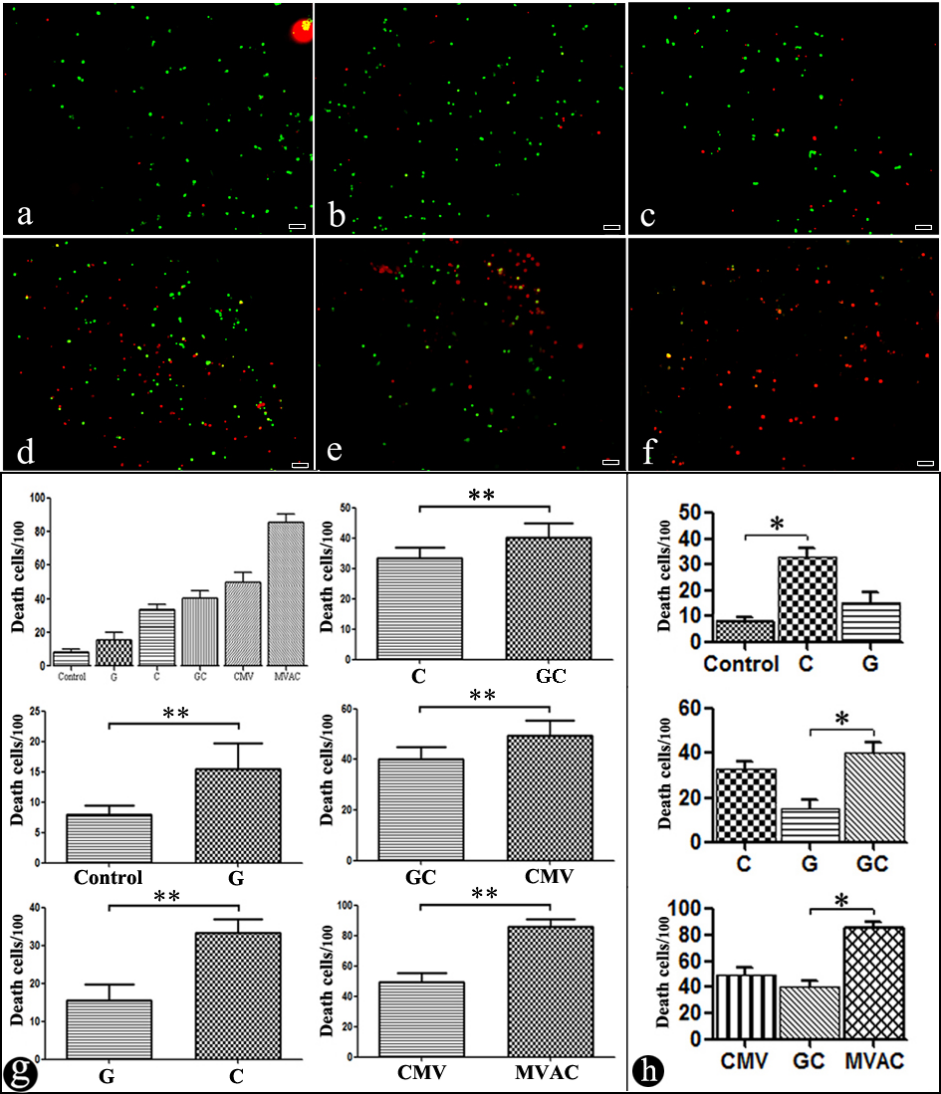
A fluorescence photograph of bladder cancer cells treated with different chemotherapy regimens **a.** Control. **b.** G (gemcitabine). **c.** C (cis-diammineplatinum dichloride). **d.** GC (gemcitabine and cis-diammineplatinum dichloride). **e.** CMV (cis-diammineplatinum dichloride, methotrexate and vincristine). **f.** MVAC (methotrexate, vincristine, doxorubicin and cis-diammineplatinum dichloride). 40×, scale bar 50 μm. **g.**, **h.** A pictograph of different chemotherapy regimens. Mean±SD. **g.** Wilcoxon rank sum-test, **p ≤ 0.05. **h.** Kruskal Wallis-test, *p < 0.01.

Generating a chemotherapeutics sensitivity assay for bladder cancer in this system is the main purpose of this research. In this study, six different chemotherapeutics regimens were used to explore bladder cell sensitivity. The chemotherapy drug concentrations were simulated based on bladder cancer patients that use chemotherapy. Cell death was evaluated using acridine orange (AO) and ethidium bromide (EB) fluorescent labeling. The chemotherapeutic schemes included gemcitabine (G), cis-diammineplatinum dichloride (C), ‘gemcitabine+cis-diammineplatinum dichloride’ (GC), ‘cis-diammineplatinum dichloride + methotrexate+vincristine’ (CMV), and ‘methotrexate + vincristine + doxorubicin + cis-diammineplatinum dichloride’ (MVAC). The chemotherapy regimens were based on clinical neo-adjuvant schemes for bladder cancer. The effect of the schemes (G/C/GC/CMV/MVAC) is reflected by the fluorescence images (Figure 7b-7f). Figure 7a shows the blank control scheme without chemotherapy drugs. Comparing the schemes (Blank vs. G, C vs. G, C vs. GC, CMV vs. GC and MVAC vs. CMV), their sensitivity differences were fully reflected using this system. (Figure 7g. Wilcoxon rank sum-test, ** p≤0.05). By comparing the single drug regimens with the control (G/C/control) and the single chemotherapy drug regimens with the combined chemotherapy drug regimens (G/C/GC), the sensitivities of the chemotherapy regimens clearly differed (Figure 7h. Kruskal Wallis-test, * p < 0.01).

## DISCUSSION

In this research, four types of cells were successfully co-cultured in a platform we constructed. The major and significant cells were selected to reconstitute a tumor microenvironment. Unlike a co-culture with two types of cells or a monoculture, in this study, more elements involved in a microenvironment were introduced into the system. A dynamic pattern for the cell-culture medium was provided through continuous perfusion with a simple column, which is a good analogy for blood flow in a tumor microenvironment. Compared with a traditional cell assay method, four types of cell morphologies and motilities were simultaneously captured in real time using this system. Moreover, this system may be combined with micro-western arrays technology to solve the problem of the system not being high throughput enough to assay the molecular signaling effects due to its limited number of cells.

As shown in Figure 4, the macrophage migration toward a bladder cancer cell (T24) in this system is a good analogy for the monocyte/macrophage recruitment process toward a neoplastic site in vivo. Related research indicates that various factors in a tumor microenvironment stimulate macrophage recruiting to tumor cells, such as chemokine ligand 2(CCL2) and macrophage colony stimulating factor (M-CSF).[15] In addition, macrophage recruitment in a tumor microenvironment is a complex process that involves biological pathways. Pallavi Chaturvedi et al. demonstrated that a hypoxia-inducible factor (HIF)-correlated signaling pathway, which involved chemokines (C-C motif) ligands and chemokine receptor type-5, drove the macrophage recruitment process in breast cancer. The HIF-correlated signaling pathway correlated macrophage recruitment and an intratumoral hypoxia environment. [16]

Phenotypic alteration of a portion of the stromal cells is a characteristic of tumor microenvironments. In this study, Figure 6 shows that Arg-1 was highly expressed in the microenvironment simulation group, which acted as a good analogy for the macrophage activation process in a tumor microenvironment in vivo. As a vital component of microenvironments, macrophages are heterogeneous and can be divided into two categories: M1 and M2 phenotypes. Arg-1 is overexpressed in the M2 phenotype macrophage and is widely used as an effector molecule to detect macrophage activated states.[17] The M1 phenotype macrophage can destroy tumor cells and participates in a normal immunoreaction. In contrast, tumor associated macrophages (TAMs) are biased toward M2 phenotype macrophages, which promote tumor progression and metastasis.[17, 18]

The ‘reticular’ structure phenomenon in bladder cancer cells (shown in Figure 5) is linked to the theory of “tumor cell vasculogenic mimicry”, which refers to de novo formation of vascular networks in the plasticity of aggressive neoplastic cells without endothelial cell participation.[19, 20] Related research has demonstrated that tumor growth and metastasis are associated with vasculogenic mimicry (VM) in bladder cancer. Bladder cancer cells can form a network, linking vascular endothelial cells, and contribute to tumor microcirculation.[21] Different expression levels for CD133 and CD82/KAI1 were correlated to VM occurrence in bladder cancer and facilitate relapse and invasion of bladder urothelial carcinoma.[22] In the clinical environment, tumor patients with negative VM exhibited a better 5-year survival rate than patients with positive VM.[23] The dynamic interaction between microenvironment and neoplastic cells has been correlated to VM formation.[11, 19] The vasculogenic mimicry phenomenon is significant in bladder cancer microenvironment simulation.

Endothelial cells are vital for forming vessels in a microenvironment and promote tumor progression and metastasis. Vessel density increases from benign tissue to a malignant transition in most solid tumors.[24] In a related study, vascular remodeling also correlated with macrophages through several biotic factors, such as CSF-1, in activating the angiogenic switch.[25] Moreover, increased lactate secretion and fluctuating oxygen concentrations, which are hallmarks of tumor cell metabolism, give rise to a fluctuating acid microenvironment with glucose consumption and provide a crucial means for tumor-associated angiogenesis through activating recruited endothelial cells.[26] Based on our research team's findings, we hypothesized that tumor cells undergo aerobic glycolysis to produce lactate, which enters endothelial cells by protein monocarboxylate transporter-1(MCT1). The increased concentration of lactate promotes angiogenesis, and endothelial cell glycolysis provides energy for angiogenesis. [27, 28]

Bladder cancer is a disease that is heterogeneous for chemosensitivity, which leads to uncertainty in the treatment effects of individual therapy. Many studies have demonstrated that tumor chemosensitivity is correlated to stromal cells in the tumor microenvironment.[29, 30] For instance, TAM can express “chemoprotective” factors, such as cathepsins B and lysosomal enzymes, which protect tumor cells from destruction by limiting the paclitaxel (PTX) therapeutic responses.[31] Paulus et al. reported that anti-colony-stimulating factor 1 (CSF1) antibodies induced TAM depletion and increased the efficacy of a combination chemotherapy with methotrexate, 5-fluorouracil and cyclophosphamide.[32] In a clinical environment, certain patients with bladder cancer and treated with a chemotherapy regimen suffer from chemotherapy drug side-effects but do not receive positive effects, which destroys the patient's physical condition, and the patients lose the opportunity to receive additional therapy.[33, 34] Thus, testing chemotherapy drug sensitivity in patients is critical and significant for individual therapy. Related chemosensitivity tests have explored the tumor cell response to different concentration gradients of chemotherapeutics, but ignore the actual drug concentration in the blood of patients treated with chemotherapeutics.[2, 35] In this research, several different chemotherapy regimens were used in this microfluidic device through simulating the chemotherapeutic concentrations in adjuvant chemotherapy patients with bladder cancer, which provides an interaction between neoplastic cells and drugs that more closely resembles the tumor microenvironment.

The future direction of this research is to promote development of “precision medicine” in tumor therapy. We aim to fabricate a biological platform to simulate tumor microenvironment and demonstrate that this simulated microenvironment can be used to test drug sensitivity in bladder cancer. Based on the test results herein, we provide a preliminary foundation for neo-adjuvant chemotherapy in bladder cancer; further, we establish a theoretical foundation for tumor microenvironment simulation using primary cultured cells and prepare to provide a platform for individual tumor therapy.

## MATERIALS AND METHODS

### Microfluidic device fabrication

The microfluidic device consisted of two layers, a polydimethylsiloxane (PDMS) piece and a glass slide. The PDMS (Sylgard 184, Dow Corning, Midland, MI, USA) piece was fabricated by replicate molding on a silicon wafer, which was patterned by photolithography after spin coating a 50-μm layer of a SU8-3025 negative photoresist (Microchem Corp., Newton, MA, USA). After mixing thoroughly, the PDMS precursor and curing agent mixture (10:1 w/w) was poured onto the mold and degassed in a vacuum chamber for 30 min. Next, the assembly was oven-cured for 2 h at 75°C. Once cured, the PDMS piece was gently peeled from the mold and trimmed to size. After creating the inlet and outlet holes, the PDMS piece was sonicated in anhydrous ethanol for 5 min and then dried in an oven. Finally, the PDMS piece was bound onto a glass substrate after 25 sec of an oxygen plasma treatment. Prior to use, the microfluidic device was sterilized with UV light for 1 h.

### Functional characterization of the microfluidic device

The matrigel matrix (BD Biocoat) was introduced into the matrigel channels through matrigel inlets. The microfluidic device was placed in a petri dish with little ultra-pure water and was maintained in an incubator at 37°C for 8 h. The microscopy images of the matrigel channels filled with a matrigel matrix are shown in Figure 2 (a1-a2). Next, two steps were performed to validate molecular diffusion using this device. In step one, a four-fold diluted matrigel matrix with culture medium was loaded into the chip inlets and driven into co-culture chambers. FITC-Dextran was introduced from the medium inlet into medium outlet. In step two, as shown in Figure 2 (c1), a four-fold diluted matrigel matrix with culture medium was loaded into the cell culture chamber B, chamber C and chamber D via the cell inlets. FITC-Dextran was then loaded into cell culture chamber A.

### Cell co-culture in the microfluidic device

In this study, human bladder cancer cells (T24), macrophages (Raw 264.7), fibroblasts (BJ-5Ta) and human umbilical vein endothelial cells (HUVECs) were used to reconstitute a bladder cancer microenvironment. The four types of cells were obtained from the cell bank of the Central Lab of The Affiliated Hospital of Qingdao University. The T24 cell line was maintained in the medium RPMI-1640 (HyClone) supplemented with 10% fetal bovine serum (HyClone) and 1% penicillin−streptomycin (Solarbio, China) at 37°C with 95% relative humidity and 5% CO_2_. The Raw 264.7 cell, BJ-5Ta cell and HUVEC cell lines were grown in DMEM medium supplemented with 10% fetal bovine serum (HyClone) and 1% penicillin−streptomycin (Solarbio, China) at 37°C with 95% relative humidity and 5% CO_2_. To obtain 2D and 3D cell suspensions, four types of cells were resuspended in the culture medium and matrigel matrix (4-fold dilution with the culture medium) at 1×10^6^ cells/ml, respectively. The T24 cells, Raw 264.7 cells, fibroblasts and HUVECs were loaded into co-culture chambers A, B, C, and D, respectively. The medium perfusion device was connected to the medium channel inlet to trigger medium flow in the medium channel (shown in Figure 1c). Cell culture medium (DMEM medium supplemented with 10% fetal bovine serum and 1% penicillin−streptomycin) was introduced into the microfluidic device inlet and driven into the medium channel using a microscale vacuum suction apparatus connected to the medium channel outlet. After the culture medium filled the whole medium channel, removing the microscale vacuum suction apparatus from the microfluidic device, the culture medium flowed slowly relying on the liquid pressure of the liquid column. Next, the assembly device was maintained in an incubator at 37°C, 95% relative humidity and 5% CO2.

To show that cell co-culture using this system can induce changes in protein molecule expression compared with the control, we used one cell type (random selection; here, we used Raw 264.7 cells) to test the different levels of relevant protein molecule expression between the microenvironment simulation and control groups.

In the microenvironment simulation group, T24 cells, macrophages (Raw 264.7), fibroblasts (BJ-5Ta) and HUVECs were introduced into chambers A, B, C and D in this system, respectively. In the control group, the macrophages (Raw 264.7) were loaded into chambers A, B, C and D. Next, a cell-culture medium perfusion device was connected to the microfluidic chips. The experimental and control group assembly devices were maintained in an incubator at 37°C, 95% relative humidity and 5% CO_2_. After co-culturing for 2 days, the macrophages were washed 3 times with PBS, fixed in 4% Paraformaldehyde and 5% glutaraldehyde for 20 min, rinsed 3 times in PBS for 10 min, permeabilized in 0.2% Triton X-100 for 3 min, and washed again with PBS. The cells were then blocked with 2% bovine serum albumin (BSA) for 60 min at room temperature and rinsed 3 times in PBS for 5 min. Next, the cells were incubated with a primary antibody (Arg-1: 1:350, ABclonal) at 37°C. After 60 min, the cells were rinsed 3 times with PBS for 5 min, incubated with a secondary antibody (TRITC- goat anti-rabbit Ig G, 1:64, Boster Inc.) for 60 min, and washed 3 times with PBS for 5 min. The cell nuclei were then stained with DAPI. The images were captured by an inverted fluorescent microscope (Nikon Eclipse Ti; Software vision: NIS-Elements F 3.2) and processed with the Image J software (Vision 2.1.4.7).[36,37]

### Drug sensitivity assay for bladder cancer cells using this system

To promote “precision medicine” development in bladder cancer therapy, a drug sensitivity test for bladder cancer cells (T24) was performed using different chemotherapeutic drugs and this system. Clinical chemotherapeutic drugs for bladder cancer, gemcitabine (G) (Aladdin, China), cis-diammineplatinum dichloride (C) (Sigma Int USA), methotrexate (M) (Aladdin, China), vincristine (V) (Aladdin, China) and doxorubicin (D) (Solarbio, China) were selected and applied in this experiment. Based on clinical chemotherapy regimens, in this study, we studied a blank control group, G (gemcitabine) group, C (cis-diammineplatinum dichloride group), GC (gemcitabine and cis-diammineplatinum dichloride) group, CMV (cis-diammineplatinum dichloride, methotrexate and vincristine) group and MVAC (methotrexate, vincristine, doxorubicin and cis-diammineplatinum dichloride) group. In addition, the drug concentrations in the patients’ blood were estimated based on clinical chemotherapeutic drug dosages from the book *Chinese Diagnosis and Treatment of Urological Diseases Guide* (version: 2014, page 46).

The components and concentrations of drugs for each group are shown in Table 1. The cell culture medium contained chemotherapeutic drugs and was introduced into the microfluidic device inlet and driven into the medium channel using a medium perfusion apparatus. Next, the assembly device was maintained in an incubator at 37°C with 95% relative humidity and 5% CO_2_. Cell death was evaluated using acridine orange (AO) and ethidium bromide (EB) fluorescent labeling. After 24 h incubation, the bladder cancer cells were stained with AO/EB (Solarbio China; AO: 2 μg/ml, EB: 2 μg/ml) for a double stain. Fluorescence images of the cells were collected using a fluorescent inverted microscope (Nikon Eclipse Ti; Software vision: NIS-Elements F 3.2) and processed using Image J (Vision: 2.1.4.7) software.

**Table 1:** The estimated concentrations of chemotherapeutic drugs in different groups based on the book Chinese Diagnosis and Treatment of Urological Diseases Guide (version: 2014; page 46)

Control	Single chemotherapy drug		Combined chemotherapy drugs		
Culture medium	Culture medium +G	Culture medium +C	Culture medium +GC	Culture medium +CMV	Culture medium +MVAC
**0 mg/ml**	**G: 0.41 mg/ml**	**C: 0.0261 mg/ml**	G: **0.41 mg/ml**	C: **0.037 mg/ml**	M: **0.011 mg/ml**
			C: **0.0261 mg/ml**	M: **0.011 mg/ml**	V: **0.0015 mg/ml**
				V: **0.0015 mg/ml**	A: **0.011 mg/ml**
					C: **0.0261 mg/ml**
